# Retrospective cohort study of fluctuations in emergency department visits for nonspecific back and neck pain during the COVID-19 pandemic

**DOI:** 10.1177/03000605241302010

**Published:** 2024-12-06

**Authors:** Nissim Ohana, Yuval Baruch, Alex Tavdi, Ezequiel Palmanovich, Daniel Benharroch, Itzhak Engel, Eyal Yaacobi

**Affiliations:** 1 Department of Orthopaedic Surgery, Meir Medical Center, Kfar-Sabba, affiliated with Faculty of Medicine, TAU, Tel Aviv, Israel; 2 Spine Surgery Unit, Meir Medical Center, Kfar-Sabba, affiliated with Faculty of Medicine, TAU, Tel Aviv, Israel; 3 Pathology, Faculty of Health Sciences, affiliated with Ben Gurion University of the Negev, Beer-Sheva, Israel

**Keywords:** Retrospective cohort study, emergency department, nonspecific back pain, nonspecific neck pain, COVID-19 pandemic, health care utilization

## Abstract

**Objective:**

We examined fluctuations in emergency department (ED) visits for nonspecific back and neck pain during the COVID-19 pandemic and explored potential contributing factors.

**Methods:**

This retrospective cohort study included patients who presented to the ED with nonspecific back and neck pain between January 2019 and December 2021. Demographic data, visit frequencies, and clinical outcomes were analyzed to assess the impact of the pandemic on visit patterns.

**Results:**

A total of 1245 ED visits were recorded. Visits decreased by 30% during the peak of the pandemic, with a gradual return to baseline by mid-2021. No significant changes in patient demographics or clinical outcomes were noted during the pandemic. However, a proportional increase in neck pain visits was observed.

**Conclusions:**

The observed decline in visits may be linked to pandemic-related concerns, such as fear of exposure in the hospital and reduced activities. The increased neck pain visits highlights the potential influence of pandemic-related stress and lifestyle changes. Visit patterns rebounded as the pandemic eased, indicating a temporary decrease unrelated to condition severity. The COVID-19 pandemic temporarily affected ED visits for nonspecific back and neck pain. Further research is needed to explore the long-term effects of the pandemic on health care utilization.

## Introduction

The COVID-19 pandemic caused substantial disruptions in health care systems globally, leading to fluctuations in patient volumes in emergency departments (EDs) worldwide. Nonspecific back pain (BP) and neck pain (NP) are common reasons for emergency visits, often linked to stress and lifestyle factors.^1^ During the pandemic, changes in daily routines, increased stress, and limited access to health care services may have influenced the incidence of BP- and NP-related ED visits. Some reports suggest an increase in these visits owing to heightened stress, although others indicate a decline, possibly driven by patients’ fear of contracting SARS-CoV-2 in health care settings.^2^

Numerous studies have established a strong link between psychological stress and the onset or exacerbation of nonspecific NP and BP.^3–7^ Psychological factors such as stress, anxiety, and depression are contributors to musculoskeletal pain, including chronic conditions. In a systematic review by Linton,^3^ stress and distress were consistently found to be associated with NP and BP across various studies. The biopsychosocial model is increasingly recognized as a comprehensive approach to understanding pain, highlighting how emotional and cognitive factors play crucial roles in both acute and chronic pain scenarios.

Nonspecific BP is especially influenced by a range of psychosocial stressors.^4^ Economic hardship, such as that experienced during a financial recession, has been shown to increase the prevalence of chronic pain conditions, including nonspecific low BP, with individuals experiencing heightened anxiety over job security and financial stability.^5^ Similarly, social stressors—such as social isolation and limited access to supportive networks—amplify emotional strain, further contributing to increased pain perception.^6^ Research has consistently demonstrated that fear-avoidance beliefs, catastrophizing, and anxiety can worsen the experience of pain, creating a feedback loop where mental stress exacerbates physical symptoms.^7^

Troubling BP or NP that has no apparent organic foundation is linked to adverse effects on individuals’ lives.^8^ This type of pain, not stemming from a defined spinal condition such as trauma, infection, tumor, or other known BP cause, is essentially experienced in the absence of an identifiable physical cause.^8–10^ Nonspecific NP, the most common cause of neck symptoms, typically arises from postural and mechanical causes but is often the subject of investigations regarding the influence of psychological stress.^11,12^

Building upon existing research demonstrating the important role of psychological factors like stress, anxiety, and mood in the development and aggravation of NP and BP,^11^ we hypothesized that the extraordinary stressors that arose during COVID-19 pandemic led to an increase in ED referrals for nonspecific BP and NP.

The interaction between psychological stress and musculoskeletal pain is not unidirectional. Research has shown that pain itself can exacerbate psychological distress, creating a feedback loop that intensifies both physical and mental symptoms. Studies^13–15^ have demonstrated that individuals who catastrophize or have higher levels of fear-avoidance behaviors are more likely to report persistent pain.

The socioeconomic impacts of NP and BP, long recognized owing to disability and work absenteeism,^16^ have been further highlighted under the unique circumstances during the COVID-19. This period offers a novel opportunity to explore how global crises can directly affect these prevalent health issues.^17^

The first year of the COVID-19 pandemic saw enormous changes aimed at curbing the spread of infection in countries worldwide. Strategies such as lockdowns were implemented, which severely limited individuals’ freedom of movement to seek support from friends and relatives.^18,19^ This issue led to limited social interaction, which further caused stressful episodes.^20^ Movement restrictions, including bans on driving, also likely led to a reduction in trauma-related ED visits, including those resulting from car accidents.^21^ These factors were considered in formulating our hypothesis regarding the patterns of ED visits for nonspecific NP or BP during the pandemic.

In Israel, strict lockdown measures were in place during the pandemic. The government enforced a stay-at-home policy, allowing people to leave only for essential purposes such as purchasing food or seeking medical help. Only individuals providing essential services were permitted to continue working and all recreational activities, including sports, were prohibited. Outpatient clinics and elective procedures were significantly reduced or postponed. These restrictions influenced individuals’ behavior, potentially affecting the flow of patients to the ED for conditions like nonspecific BP and NP.

In this comparative analysis, we sought to elucidate the complex interplay between psychological stressors and the manifestation of physical pain symptoms, particularly in an era defined by a global health crisis.^22^ The findings can potentially inform future health care strategies and interventions, especially regarding the management of psychosocial factors in pain perception and treatment.

Our fundamental hypothesis posits that the incidence of individuals seeking ED treatment for nonspecific BP and NP did not vary significantly between the pre-pandemic period and the COVID-19 pandemic period, barring any unique influences attributable to the pandemic itself. The pandemic emerged at the start of 2020, at which time regional authorities implemented a social isolation strategy to curb virus transmission among the population.^23^

In a previous preprint publication,^24^ we initially reported our findings regarding the impact of COVID-19 lockdowns on ED visits for nonspecific BP and NP. The aim of this study was to assess fluctuations in BP- and NP-related visits to the ED before and during the COVID-19 pandemic using data from a large public hospital. In this analysis, we sought to determine whether the pandemic led to significant changes in the patterns of ED presentations for these conditions.

## Methods

This retrospective cohort study, conducted at a single institution in Israel, was approved by the Internal Review Board (no. MMC-0324-20; 5/1/2021) and performed in accordance with the Declaration of Helsinki of 1975, as revised in 2013. Because this was a retrospective study, obtaining signed consent from patients was not required. Patients were selected consecutively in the order of their presentation to the ED for nonspecific BP and NP during the study period. The reporting of this study adheres to the STROBE (Strengthening the Reporting of Observational Studies in Epidemiology) guidelines.^25^

In the study, we used a historical control to make comparisons of ED visits for nonspecific BP and NP. All Israeli citizens have access to a public health care service through government-mandated health insurance. Our hospital, located in an urban area, serves a diverse population and functions as a regional trauma center, handling both routine medical care and emergency cases, including trauma-related incidents. The hospital has a capacity of over 900 beds and provides comprehensive medical services across various specialties. This setting allows for a wide spectrum of patients, ranging from younger adults to older individuals, contributing to the relevance of the study findings.

To categorize visits to the ED for nonspecific BP or NP, we selected diagnoses from the International Classification of Diseases, Tenth Revision (ICD-10) indicating a nonspecific source of spinal pain. We compiled all relevant ICD-10 diagnoses that might reflect nonspecific pain and organized them into three categories: nonspecific BP, nonspecific NP, and other nonspecific pain. The third category included diagnoses that could potentially become specific at a later time. However, after reviewing the patient data, we confirmed that patients categorized as having “other” types of pain were not admitted later with a specific diagnosis of back or neck disease and did not undergo any advanced investigations for these conditions. Table 1 shows the ICD-10 diagnoses applied in this study.

**Table 1.** International Classification of Disease, Tenth Revision (ICD-10) codes for the diagnosis of back and neck pain.

ICD-10 code	Diagnosis
Non-specific back pain (BP)	
M54.9	Backaches
M54.9	Other and unspecified disorders of back
M54.5	Low back pain (Lumbago)
M54.6	Pain in thoracic spine
M54.9	Dorsalgia, unspecified
M53.3	Sacrococcygeal disorders, not elsewhere classified
M54.89	Other dorsalgia
M99.03	Segmental and somatic dysfunction of lumbar region
Non-specific neck pain (NP)	
M54.2	Cervicalgia/neck pain
M50.9	Cervical disc disorder, unspecified
M53.1	Cervicobrachial syndrome
M54.81	Occipital neuralgia
M43.6	Torticollis, unspecified
M54.2	Cervicalgia
M54.2	Neck pain
M53.0	Other disorders of cervical region
M54.2	Cervical pain
M47.812	Spondylosis without myelopathy or radiculopathy, cervical region
Other	
M51.36	Other intervertebral disc degeneration, lumbar region (Degeneration of lumbar or lumbosacral intervertebral disc)
M51.26	Other intervertebral disc disorders
M51.36	Degeneration of intervertebral disc
M48.02	Spinal stenosis in cervical region
M46.1	Sacroiliitis, not elsewhere classified
M51.34	Other intervertebral disc degeneration, thoracic region

### Exposure and outcome analysis

In our study, the time frame was methodically divided into two calendar-matched periods: 30 weeks after the initiation of lockdown owing to the COVID-19 pandemic (March to October 2020) and 30 weeks prior to the commencement of lockdowns (March to October 2019). This approach ensured a comparative analysis between equivalent time spans. The primary outcome measure in our research was the mean weekly number of patients newly diagnosed with nonspecific BP or NP.

#### Inclusion and exclusion criteria

- In this study, we included patients who presented to the ED with a first episode of NP or BP; those with severe underlying conditions; patients who were not hospitalized and did not undergo advanced investigations; and those who were discharged with no specific recommendations and who were not readmitted to the hospital later.
- We excluded patients with ED referrals attributed to a specific cause or related to trauma; those who were later admitted with a specific diagnosis indicating back or neck disease; patients who underwent advanced investigations for BP or NP; those who were re-admitted owing to specific spinal problems; and patients under the age of 18 years.

We delineated two distinct time frames for the analysis: a period during the first year of the COVID-19 pandemic and a comparable period in the calendar year prior to the start of the pandemic. We investigated the frequency of ED visits each week during each of these periods. Additionally, we analyzed demographic variables, such as age and sex, as well as variables specific to the emergency visit itself. Patients included in the present research received treatment in the ED and were negative for SARS-CoV-2 in reverse transcription polymerase chain reaction testing.

### Statistical analysis

To characterize continuous and categorical variables, we used number with percentage and mean with standard deviation, respectively. Differences in demographic data between the two groups (pre- and post-lockdown periods) were assessed using analysis of variance.

In the present analysis, β_0_ represents the initial level of weekly ED presentations 1 year before lockdowns began in Israel (serving as the intercept). β_1_ quantifies the pre-lockdown trend of weekly ED presentations. Β_2_ indicates the level change at the first lockdown point, attributable to the influence of lockdown. Β_3_ is an estimate of the change in slope from before to after the start of lockdowns, reflecting the variance between pre- and post-lockdown trends. All statistical analyses were conducted using SAS version 9.4 (SAS Institute Inc., Cary, NC, USA), with a two-sided p-value of <0.05 set as the threshold for statistical significance.

## Results

**Figure 1.**
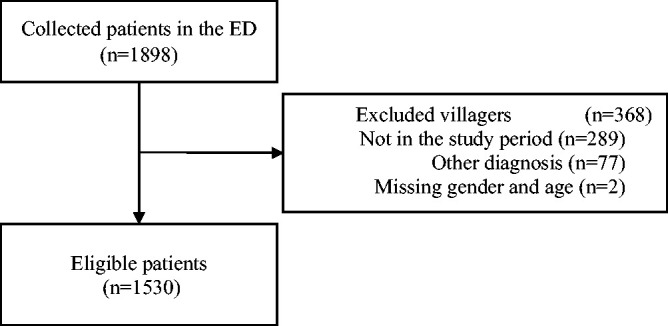
Flow diagram of eligible patients.

In the present comprehensive hospital database analysis, we identified 1898 visits to our ED for nonspecific BP and NP between March 2019 and October 2020. The lockdown periods were within this time frame. From the total cohort, 368 patients were excluded for various reasons, including non-attendance during the study period, alternative specific diagnoses, reclassification of nonspecific pain to specific pain after further investigation, or incomplete data, as shown in the flow diagram (Figure 1). This resulted in a final study sample of 1530 patients with ED visits for possible nonspecific BP or NP (Table 2).

**Table 2.** Baseline characteristics of eligible patients by diagnosis.

Variable	Low back pain/sciatica(n = 1090)	Neck pain/cervicalgia(n = 440)	p value
Sex, n (%)			0.30
Male	582 (72.4)	222 (27.6)	
Female	508 (70.0)	218 (30.0)	
Age (y), mean (SD)	52.10 (16.97)	46.64 (18.45)	<0.001
Age group (y), n (%)			<0.001
<20	3 (15.0)	17 (85.0)	
20–39	260 (63.0)	153 (37.0)	
40–59	468 (73.4)	170 (26.6)	
≥60	359 (78.2)	100 (21.8)	
Time in ED (h), mean (SD)	3.79 (1.93)	3.58 (1.99)	0.02
Time in ED (h), n (%)			0.004
<2 h	165 (63.7)	94 (36.3)	
≥2 h	925 (72.8)	346 (27.2)	
Lockdown, n (%)			<0.001
Before	801 (74.7) (95% CI: 72.0–7.3)	271 (25.3) (95% CI: 22.7–8.0)	
During	289 (63.1)(95% CI: 58.5–67.6)	169 (36.9)(95% CI: 32.4–1.5)	

ED, emergency department; SD, standard deviation.

Table 2 shows a comparison of patient demographics and presentation details between patients with BP (n = 1090) and NP (n = 440). No significant sex differences were noted, with a higher incidence of BP found among men (72.4%) compared with women (70.0%). In contrast, NP was nearly equally distributed between male (27.6%) and female (30.0%) patients. Notably, patients over age 60 years were predominantly affected by BP whereas NP was more common in the age group 40–59 years. Additionally, most patients with BP experienced a prolonged stay in the ED exceeding 2 hours.

A combined analysis revealed a decrease in the rate and number of BP-related ED visits during the lockdown period to 289 (63.1%; 95% confidence interval [CI]: 58.5–67.6) from 801 (74.7%; 95% CI: 72.0–77.3) in the year preceding the pandemic. In contrast, although the absolute number of NP-related visits declined from 271 to 169, the rate increased from 25.3% (95% CI: 22.7–28.0) to 36.9% (95% CI: 32.4–41.5). This indicates a notable shift in patient behavior and decision-making regarding visits to the ED during the pandemic.

Table 3 presents a comparative analysis of weekly ED visits before and during the pandemic. Significant variance was observed in mean ED visits between these two periods (p < 0.001). The median weekly visits stood at 36.5 before the pandemic, which declined to 16 during the pandemic period, depicting the effects of the pandemic on the use patterns of health care services.

**Table 3.** Weekly number of ED presentations before and during lockdowns.

	Before lockdown	During lockdown	p value
Weekly ED presentations			<0.001
Mean (SD)	37.1 (7.1)	16.5 (5.7)	
Median (IQR)	36.5 (31.0, 42.0)	16.0 (25.0, 50.0)	

ED, emergency department; SD, standard deviation; IQR, interquartile range.

Table 4 provides a detailed statistical account, revealing a substantial decrease in weekly ED visits at the onset of lockdowns (mean difference = −22.2, 95% CI = −28.7 to −15.7, p < 0.001). Notably, the trend in weekly visits did not show a significant difference when comparing the period before the pandemic with that during lockdowns (slope difference = 0.19, 95% CI = −0.08–0.45).

**Table 4.** Interrupted time-series analysis for the weekly number of presentations.

	ED presentations (per week)		
	Coefficient	95% confidence interval	P value
Presentations			
Intercept (β_0_)	38.5	33.8–43.2	<0.001
Trend before lockdown (β_1_)	−0.09	−0.36–0.18	0.51
Level change after lockdown (β_2_)	−22.2	−28.7–−15.7	<0.001
Trend after lockdown (β_3_)	0.28	−0.10–0.65	0.16
Trend change after lockdown (β_1_ + β_3_)	0.19	−0.08–0.45	0.17

ED, emergency department.

Patients’ demographic and clinical characteristics before and during lockdowns are summarized in Table 4. A consistent pattern of more male patients seeking ED care during both periods was observed. Interestingly, the average age of patients was older prior to the pandemic, and the length of stay in the ED was longer before the initiation of lockdowns. The data also revealed a distinct pattern in NP and BP incidence. Although the number of visits for these conditions decreased during lockdowns, the proportion of ED visits attributable to NP rose from 24.4% to 34.1% (Table 5)

**Table 5.** Comparison of patient demographics and clinical characteristics before and during COVID-19 lockdowns.

Variable	Before lockdowns (25 March 2019–21 October 2019)	During lockdowns (25 March 2020–21 October 2020)	p value
Sex			Chi-square test
N (Nmiss)	1113 (1)	494 (1)	0.01
M	563 (50.6%)	284 (57.5%)	
F	550 (49.4%)	210 (42.5%)	
Age, y			*t-*test
N (Nmiss)	1113 (1)	494 (1)	0.01
Mean (SD)	50.39 (18.53)	47.84 (18.32)	
Median	49.00	48.00	
Q1; Q3	38.00; 65.00	35.00; 61.00	
Min; Max	2.00; 98.00	0.00; 92.00	
Length of stay (h)			*t-*test
N (Nmiss)	1114 (0)	495 (0)	0.003
Mean (SD)	3.80 (2.01)	3.50 (1.82)	
Median	3.48	2.23	
Q1; Q3	2.38; 4.82	2.27; 4.43	
Min; Max	0.38; 16.50	0.43; 15.43	
Diagnosis in ED			Chi-square test
N (Nmiss)	1114 (0)	495 (0)	<0.0001
Back pain	801 (71.9%)	290 (58.6%)	
Neck pain	272 (24.4%)	169 (34.1%)	
Other	41 (3.7%)	36 (7.3%)	

Nmiss, number of missing patients or cases for a particular variable or dataset; ED, emergency department; SD, standard deviation.

**Figure 2.**
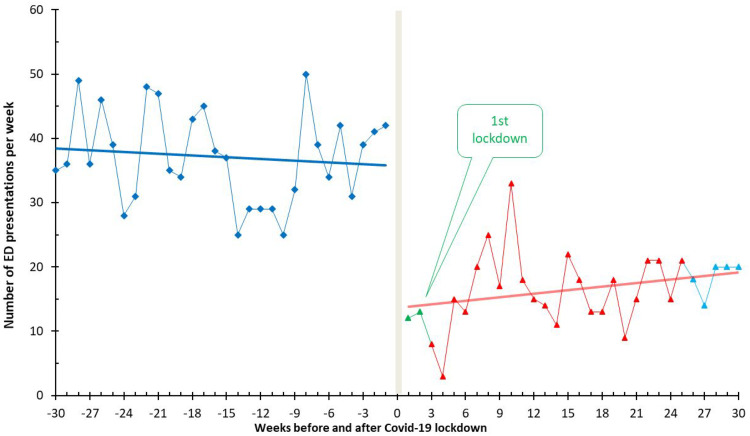
Weekly number of emergency department presentations among eligible patients before and during lockdowns.

The first quartile of the pandemic witnessed a significant drop in weekly presentations to 25 per week, compared with 31 in the previous year. In the third quartile, coinciding with the easing of lockdown measures, the average number of ED visits increased to 50 per week, equating to the count observed in the preceding year. This trend is further illustrated in Figure 2, which depicts fluctuations in the mean number of ED presentations each week across the two study periods. The onset of lockdowns was marked by a steep decline in weekly emergency visits, followed by a moderate increase upon easing of restrictions.

Table 6 outlines the results of multivariable regression analysis exploring factors associated with the 1090 cases of BP diagnosed in the ED. Sex did not emerge as a significant factor. The age group most significantly affected was between 20 and 40 years (p < 0.001). The age groups <20 (p < 0.001) and 40 to 59 years (p = 0.05) were also significantly affected. The length of stay in the ED typically exceeded 2 hours, with a higher frequency and rate of BP cases observed before the pandemic.

**Table 6.** Multivariable logistic regression for visits among 1090 patients diagnosed with LBP in the ED.

Variable	Low back pain/sciatica(n = 1090)	Odds ratio (95% CI), p
Sex, n (%)		
Male	582 (72.4)	1.00 (ref)
Female	508 (70.0)	0.81 (0.64–1.02), 0.07
Age group (y), n (%)		
<20	3 (15.0)	0.06 (0.02–0.20), <0.001
20–39	260 (63.0)	0.47 (0.34–0.63), <0.001
40–59	468 (73.4)	0.75 (0.57–1.00), 0.05
≥60	359 (78.2)	1.00 (ref)
Time in ED (h), n (%)		
<2 h	165 (63.7)	1.00 (ref)
≥2 h	925 (72.8)	1.37 (1.02–1.83), 0.04
Lockdown, n (%)		
Before	801 (74.7)	1.00 (ref)
During	289 (63.1)	0.59 (0.46–0.75), <0.001

ED, emergency department; CI, confidence interval; LBP, low back pain.

## Discussion

The COVID-19 pandemic had adverse impacts on people worldwide.^26^ In a concise time frame, it became crucial to unravel the mechanisms of disease spread, initiate preventive strategies to thwart widespread viral transmission, and devise effective treatment protocols for infected individuals, all while safeguarding their close contacts from contracting the virus. Social isolation was a critical strategy applied across the globe to address the health crisis.^27^ Despite controversies in the use of this approach, it resulted in notable changes regarding management of the disease. The requirement for social isolation meant that individuals were barred from leisure activities, large social interactions, educational pursuits, and regular work activities, excluding essential services.^22^ However, this regime proved challenging as individuals were confined to their homes and deprived of social and physical engagement. Further, people’s lives were affected by narratives in the media that the virus was lethal and lacked any effective treatment or vaccine. These conditions further intensified health issues could have arisen among people susceptible to the effects of anxiety and stress induced by isolation and facing an unknown future.^28–32^ In our study, we investigated the prevalence of nonspecific BP and NP, using these as a proxy to explore the correlations among health impacts attributable to stress-laden environments. Linked to a sedentary lifestyle and heightened anxiety, nonspecific pain in the neck and back manifests as discomfort in the lumbar spine without an identifiable cause.^31^ This contrasts starkly with specific spinal pain, which is attributable to identifiable pathological conditions such as disc herniation, spinal stenosis, vertebral infection, or neoplasm.

A public health crisis like the COVID-19 pandemic can have significant effects on pain perception, which in turn affects both physical and mental health,^4–7^ especially among certain groups such as health care workers. Burnout syndrome, heightened anxiety, and stress, such as that present during the COVID-19 pandemic. are strongly correlated with increased musculoskeletal pain, including nonspecific NP and BP. Karlıbel et al.^33^ demonstrated that health care workers experienced high levels of emotional exhaustion and depersonalization during the pandemic, which were closely linked to BP and NP. Similar trends were observed during previous health crises like SARS and MERS, where increased psychological stress among health care workers contributed to a rise in musculoskeletal pain. These findings suggest that the fluctuations in pain perception observed during the COVID-19 pandemic are not entirely unique but part of a broader phenomenon that can occur during prolonged periods of a public health emergency in which psychological distress has a crucial role in the manifestation of physical pain symptoms. Notably, nonspecific pain is susceptible to exacerbation under heightened stress, drastic reductions in physical activity, and unexpected weight fluctuations over time.^9,10,12^

Given that BP and NP represent a frequent cause for ED visits,^32^ we postulated that the onset of the pandemic might be correlated with a shift in the patterns of these visits, possibly reflecting changes in the underlying etiology of such pain syndromes. Our research focused on investigating whether the COVID-19 pandemic had an impact on the frequency of ED visits for nonspecific BP or NP, which can be indicative of underlying mental stress.^10^ The starting assumption was that when comparing visits for the same problem during the same two periods in 2 consecutive years, there should not be a notable difference in the visit rates to the ED. This assumption is based on the idea that the fundamental occurrence of nonspecific BP or NP should remain relatively stable, even during a public health emergency.

Several factors were considered in seeking to understand the unique circumstances of the COVID-19 pandemic. Fewer emergency visits might be expected owing to reduced levels of physical activity, work, and social interaction, with people much more likely to stay at home and experiencing decreased physical strain and reduced opportunities for physical injury.^18,19^ However, we also considered the potential impact of heightened mental stress during the pandemic.^17^ Increased stress levels, resulting from factors such as social isolation,^34–36^ economic uncertainty, and health-related anxiety, could potentially manifest as physical symptoms, including nonspecific BP or NP.^11^ Therefore, an unexpected increase in the rate of emergency visits for these conditions during the pandemic would suggest the presence of a new influencing variable—a stressogenic factor. By comparing emergency visit frequencies during the pandemic to the pre-pandemic period, we aimed to determine if increased mental stress led to more ED visits. This approach tested the hypothesis that nonspecific BP or NP could indicate population stress levels.

### Shifts in emergency visit patterns for BP and NP

As described in Table 2, the percentage of BP-related visits decreased post-lockdown from 74.7% (95% CI: 72.0–77.3) to 63.1% (95% CI: 58.5–67.6), and NP-related visits increased from 25.3% (95% CI: 22.7–28.0) to 36.9% (95% CI: 32.4–41.5). These CIs indicate that the differences observed are not only statistically significant but also precise, reflecting a meaningful shift in the distribution of visits. This indicates a notable shift in patient behavior and decision-making regarding ED visits during the pandemic. The reduction in BP visits suggests that many individuals with BP deferred seeking care, possibly managing their symptoms at home owing to fear of exposure to SARS-CoV-2. Conversely, the relative increase in NP visits suggests that NP, potentially exacerbated by pandemic-related stress and lifestyle changes,^11,37,38^ became a more prominent concern requiring emergency care. Table 6 outlines the findings of multivariable regression analysis exploring factors associated with BP diagnosed in the ED. Sex did not emerge as a statistically significant factor, reinforcing that BP affects men and women equally in emergency care visits. The age group predominantly affected was between 20 and 40 years, suggesting that younger adults were also significantly impacted by BP. The length of stay in the ED typically exceeded 2 hours, highlighting the resource-intensive nature of managing BP in emergency settings. The higher frequency and rate of BP cases observed before the pandemic further emphasize the impact of lockdown measures on health care-seeking behavior.

### Impact of the pandemic on emergency visits

As detailed in Table 3, our analysis revealed a significant reduction in the mean number of weekly emergency visits for NP and BP over the pre-pandemic and pandemic periods. Median weekly visits decreased markedly from 36.5 before the pandemic to 16 during the pandemic. This sharp decline underscores the profound impact of the pandemic on health care utilization patterns, as previously described.^39,40^ Several factors likely contributed to this reduction, including the widespread imposition of social isolation measures, fear of contracting the virus in health care settings, and changes in public behavior regarding seeking medical care during the pandemic.^41^ Table 4 provides a detailed statistical account, revealing a substantial decrease in weekly emergency visits at the onset of lockdowns. The mean difference in weekly visits was −22.2, with a 95% CI of −28.7 to −15.7 and a p-value <0.001. This statistically considerable decline highlights the immediate effect of lockdown measures on reducing the frequency of emergency visits. The data suggest that patients were either unable or reluctant to visit the ED during the stringent lockdown period, likely owing to restrictions on movement and concerns about exposure to COVID-19 in hospital environments. Interestingly, although the initial decrease in weekly emergency visits was significant, the trend in weekly visits did not show an important difference when comparing the period before the COVID-19 pandemic with that after the initiation of lockdowns. The slope difference was 0.19, with a 95% CI of −0.08 to 0.45 and a p-value of 0.17. This indicates that after the initial drop, the rate of change in weekly emergency visits remained relatively stable. The stability in the trend suggests that once the population adjusted to the new normal of the pandemic, the frequency of emergency visits for nonspecific NP and BP stabilized, albeit at a lower level than that before the pandemic.

### Demographic patterns and diagnostic trends

The demographic analysis, summarized in Table 5, revealed several noteworthy patterns. No significant sex differences were noted between patients with NP and those with BP. In our sample of patients in the ED, the proportion of BP-related visits was slightly higher among men (72.4%) than women (70.0%), although this difference was not statistically significant. NP-related visits were similarly distributed between men and women. It is important to note that these percentages reflect patients presenting to the ED, not general population incidence or prevalence estimates. Therefore, whereas these patterns offer insights into ED presentations, they should not be interpreted as broader epidemiological trends in the population. This lack of a significant sex difference suggests that both conditions affect men and women seeking emergency care equally. However, Braden et al.^42^ and Von Korff et al.^43^ published findings indicating that women are more affected by BP. Additionally, Guez^37^ found that women are more affected by NP than men.

Notably, patients over the age of 60 years were predominantly affected by BP whereas NP was more common in the age group 40 to 59 years. This aligns with the literature,^44,45^ indicating that older patients are more vulnerable to BP, especially if they have comorbidities such as depression or chronic somatic diseases. The prevalence of BP among older adults illustrates that the age distribution factor, owing to chronic and degenerative changes, may impact the prevalence of this condition.^45^

Compared with BP, NP may be more related to sedentary lifestyles and instances of stress, including stress in the workplace, all of which are prevalent in middle-aged adults. According to Fanavoll et al.,^46^ work stress is an independent predictor of chronic neck or shoulder pain, with the effect being stronger in men than that in women. Physical exercise does not substantially reduce the risk among those frequently exposed to work stress. Additionally, many patients with BP had stays in the ED exceeding 2 hours. This reflects the severity and complexity associated with BP. A possible reason for longer ED stays is immobility caused by severe pain, necessitating prolonged and effective pain relief treatment before patients can be discharged.

### Initial public response to the COVID-19 pandemic

Initially, there was a lack of clear information about the typical symptoms of COVID-19, and the public was informed that almost any symptom could be a potential manifestation of the disease.^47^ This uncertainty likely contributed to a natural fear, especially among adults, leading them to seek medical attention for symptoms such as NP and BP out of concern that these could be related to COVID-19. This initial surge in emergency visits could reflect a heightened vigilance and fear among the population, which eventually stabilized as more specific information about COVID-19 symptoms became available and public understanding improved. Our results revealed significant fluctuations in the number of NP and BP emergency visits throughout the pandemic, as depicted in Figure 2. During the first quartile of the pandemic, there was a notable drop in weekly presentations to 25 per week, compared with 31 per week in the same period the previous year. The decline is related to measures for managing COVID-19 during the lockdown period. Uncertainties and public fears about the virus could also have led to the initial decline.^23^ Many individuals avoided care facilities to minimize exposure to the virus.^26^ However, the rates and trends of infection with SARS-CoV-2 shifted in the third quartile, with a number equating to that in the preceding year and with increased emergency visits of up to 50 per week. This was in line with the easing of infection control measures and the lifting of restrictions to where people were less fearful when seeking medical attention and could address any medical concerns they neglected during the period of lockdown.

### Implications for health care utilization

Weekly ED visits declined steeply during the start of lockdowns. There was also a moderate increase in such visits when restrictions were eased. This reveals the significant effects of measures related to public health on patterns in health care service utilization. From our observation, public fear and lockdowns were associated with an increased reduction in the number of patients who visited the ED, even in situations requiring an emergency visit.^21^ Avoidance behavior leads to delays in treating emergency conditions.^48^ The diagnostic and demographic trends show the importance of adaptive clinical practice and strategies in public health. Vigilance is critical among care providers when there are increases in the number of NP cases, especially regarding mental health concerns owing to pain. Health care professionals should also recognize that anxiety and stress may manifest as physical symptoms.^22^ Integrating mental health support and physical health care, particularly for conditions like NP that are susceptible to stress, could improve patient outcomes.

Targeted public health messages are also essential to reassure the public, especially older populations, about the safety of seeking medical care during a public health crisis to ensure that patients do not delay necessary treatments owing to fear of infection. Strategies to manage and mitigate stress, promote physical activity, and encourage proper ergonomics in home and remote work settings could help reduce the incidence of stress-related conditions like BP and NP.

## Study limitations

Several limitations should be acknowledged despite the insights revealed in relation to BP and NP emergency visits during the COVID-19 pandemic. The retrospective study design and reliance on ICD-10 codes may not have captured all nuances of nonspecific pain diagnoses. Use of administrative data limited our ability to verify the accuracy and completeness of the clinical information recorded. Additionally, excluding patients with incomplete data or those receiving a specific diagnosis after the initial presentation may have introduced selection bias, potentially affecting the generalizability of our findings.

The study is limited by its single-center design, which may not reflect the experiences of other hospitals or regions with different patient demographics, health care systems, or pandemic responses. In essence, the study findings cannot be generalized to settings with varied health care access levels.

External factors, including variations in public health messaging, changes in health care-seeking behavior, and evolving information regarding symptoms related to COVID-19, could have influenced the results of this research. The study was conducted during the pandemic period, with each period having different levels of public awareness and restrictions. This made it challenging to attribute the trends observed to measures related to lockdown.

The last limitation was that we failed to account for aspects such as mental health status, underlying chronic conditions, and socioeconomic factors, which may have influenced pain presentation and the decision to seek ED care. In the future, studies should focus on these factors to provide a comprehensive understanding of adverse effects of the pandemic on using health care services.

## Conclusion

The COVID-19 pandemic may have significantly impacted ED visits for nonspecific NP and BP, with an initial decline likely owing to prevention and control restrictions and public fear. Our study suggested that BP was more common in older adults, and NP may have increased in middle-aged individuals, possibly linked to lifestyle changes such as remote work and reduced physical activity. These findings highlight the need for health care models that integrate physical and mental health support during a public health crisis. Future research should further explore the potential connection between mental stress and physical health to enhance care strategies during a global health emergency.

## Data Availability

The data that support the findings of this study are stored within the hospital records and are not publicly available owing to legal and confidentiality restrictions. Access to these data is limited to authorized personnel within the institution. As such, the data cannot be shared outside the institution.
